# Influence of Population Demography and Immunization History on the Impact of an Antenatal Pertussis Program

**DOI:** 10.1093/cid/ciw520

**Published:** 2016-11-02

**Authors:** Patricia Therese Campbell, Jodie McVernon, Peter McIntyre, Nicholas Geard

**Affiliations:** 1 Melbourne School of Population and Global Health, University of Melbourne; 2 Infection and Immunity, Murdoch Childrens Research Institute, Royal Children's Hospital; 3 Peter Doherty Institute for Infection and Immunity, University of Melbourne, Parkville, Victoria; 4 National Centre for Immunisation Research and Surveillance, Children's Hospital at Westmead, New South Wales, Australia

**Keywords:** pertussis vaccine, disease transmission, antenatal vaccination, immunity, computer simulation

## Abstract

***Background.*** Antenatal pertussis vaccination is being considered as a means to reduce the burden of infant pertussis in low- and middle-income countries (LMICs), but its likely impact in such settings is yet to be quantified.

***Methods.*** An individual-based model was used to simulate the demographic structure and dynamics of a population with characteristics similar to those of LMICs. Transmission of pertussis within this population was simulated to capture the incidence of infection in (1) the absence of vaccination; (2) with a primary course only (three doses of diphtheria, tetanus, and pertussis vaccines [DTP3] commencing in 1985, 1995, or 2005 at 20%, 50%, or 80% coverage); and (3) with the addition of an antenatal pertussis program.

***Results.*** Modeled annual incidence averaged over the period 2015–2024 reduced with increasing DTP3 coverage, regardless of the year childhood vaccination commenced. Over the same period, the proportion of infants born with passive protection did not change substantially compared with the prevaccination situation, regardless of DTP3 coverage and start year. We found minimal impact of antenatal vaccination on infection in all infants when mothers were eligible for a single antenatal dose. When mothers were eligible for multiple antenatal doses, incidence in infants aged 0–2 months was reduced by around 30%. This result did not hold for the full 0- to 1-year age group, for whom antenatal vaccination did not reduce infection levels.

***Conclusions.*** While antenatal vaccination could potentially reduce infant mortality in LMICs, broader gains at the population level are likely to be achieved by focusing efforts on increasing DTP3 coverage.

There is limited information about the disease burden from pertussis globally. The most recent estimate of deaths from pertussis, largely limited to administrative data, is 60 000 deaths in children <5 years old, the great majority in low- and middle-income countries (LMICs) [1]. Clinical cases of pertussis are grossly underreported in LMICs, due to less developed surveillance systems, reduced access to healthcare services, and limited diagnostic testing [2]. Although estimated worldwide coverage of the primary course of pertussis vaccine was around 86% in 2014 [3], coverage in many LMICs, or regions within them, is much lower [2]. While worldwide pertussis vaccination coverage has substantially improved since the inception of the Expanded Programme on Immunization (EPI) [3], later adoption of vaccination in LMICs, with variable coverage due to supply and delivery issues and political instability, is likely to have led to heterogeneity in disease burden [4]. Antenatal pertussis vaccination, which could possibly be delivered in LMICs at higher coverage levels than infant doses, may offer an alternative to reduce the burden of disease in settings with intractably low EPI coverage and/or timeliness. While antenatal vaccination has provided improved pertussis control in settings with long-standing, high-coverage childhood vaccination programs [5, 6], the likely impact of antenatal vaccination in LMICs has not yet been quantified.

We have previously studied pertussis resurgence and maternal vaccination programs in settings with long-standing, high-coverage childhood vaccination programs [7, 8]. In our study of maternal pertussis vaccination, we used an individual-based model of pertussis transmission, incorporating household structure and calibrated to Australian conditions, as representative of high-income countries, to investigate the drivers of pertussis resurgence and we compared antenatal and postnatal pertussis vaccination strategies under a number of different delivery options [8]. The model found that risk of early infant cases was increased by declining maternal immunity, due to reduced opportunities for natural boosting of immunity arising from high vaccination coverage. These findings reflected the experience of countries using postnatal and antenatal vaccination [5, 6, 8, 9], where the passive direct protection provided to infants by antenatal vaccination led to substantially greater reductions in severe early infant pertussis than achieved by postnatal vaccination.

In this study, we have extended our previous work to settings with less mature vaccination programs. We have simulated our individual-based model of pertussis transmission to estimate the impact of antenatal pertussis vaccination in settings characterized by higher fertility rates and larger household sizes, on the background of lower historical vaccine coverage and differing assumptions about current levels of uptake, derived from the broad diversity of scenarios observed in the field. Assumptions regarding likely achievable antenatal coverage are derived from existing experience of maternal tetanus programs [10]. This extension of our existing model was designed to inform considerations around implementation of antenatal vaccination programs in LMICs.

## METHODS

### Demographic Model

We model the demographic structure and dynamics of a population using a previously developed individual-based model that characterizes individuals by their age, sex, and the household to which they belong [11, 12]. Using stochastic simulation, we track the life events occurring to these individuals: birth, death, couple formation and dissolution, and leaving home. The model is parameterized to broadly represent the demographics of a low- and/or middle-income country, with high fertility rates resulting in a population characterized by a higher growth rate (2.5%), younger average age, and larger average household size compared with that of a more developed country. The population of a given country will be uniquely shaped by its own demographic trajectory, and we have not attempted to model a specific country scenario, but rather to investigate the influence of demographic trends shared by multiple LMICs. Further detail is provided in the Supplementary Materials.

Despite the importance of population mixing to the transmission of infectious diseases, only a small number of studies have been published that attempt to measure age-related rates of contact between individuals. Social contact surveys focused on European settings found high levels of mixing between people of the same age (age-assortative mixing) and between parents and children (intergenerational mixing) [13, 14]. Recent studies undertaken in Zambia and South Africa [15], Vietnam [16], and Kenya [17], while differing in their methodologies and the reported number of contacts, showed that key patterns of age-assortative and intergenerational mixing remain similar across different settings. Our model simulates patterns of contact behavior that reflect the age and household structure of the population and the observed tendency for contact to occur among individuals of similar ages.

### Epidemiologic Model

#### Population Model of Infection and Immunity

**Figure 1.**
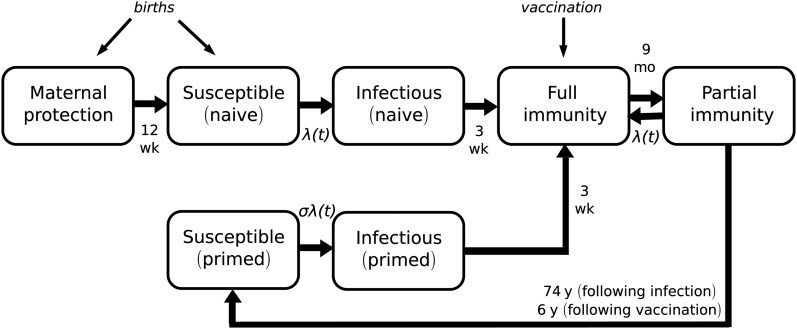
Pertussis transmission model. Infants born to immune mothers (full immunity or partial immunity) start their lives with maternal protection and are fully protected against infection, before waning into the naive susceptible state. Naive individuals are infected at a rate *λ*(*t*), and are fully protected on recovery (full immunity), before waning into a partially immune state from which their immunity can be boosted (partial immunity). If not reexposed, individuals wane to a primed susceptible state (susceptible [primed]) in which their rate of infection, *σλ*(*t*), is reduced compared to naive susceptibles (σ = 0.6). If infected from this primed state, individuals are less infectious than those experiencing their first infection (infectious [primed]). Successfully vaccinated individuals acquire full immunity and thereafter follow the same-state transitions as those with immunity acquired following exposure, although with a reduced mean duration of protection. Mean durations in infectious and immune states are shown, with details provided in Table 1.

We apply the same pertussis-specific transmission model (Figure 1) to the population as used in previous work [8]. In addition to their demographic characteristics, we also track the current state of infection or immunity of each individual.

Individuals who are naively susceptible to pertussis (Figure 1, susceptible naive), having never been infected or vaccinated, become infected (infectious naive) with a probability determined by their risks of household and community acquisition. These risks depend on an individual's age-specific community mixing patterns, and the presence of infected individuals in their household and the wider community. Once recovered, individuals are fully protected against infection (full immunity).

Over time, an individual's immunity wanes (partial immunity). Individuals who are exposed to infection at this stage have their immunity boosted at a rate equivalent to the force of infection, and regain full protection against infection (full immunity) without contributing to the force of infection. Individuals whose immunity wanes fully without being reexposed and boosted become susceptible once again (susceptible primed), with a reduced susceptibility to infection compared to their first infection. If reinfected (infectious primed), these individuals are presumed to be less infectious than those experiencing their first infection. As priming is assumed to permanently modify infection risk and characteristics [18], the model distinguishes between those naively susceptible, and those with immune systems primed by pertussis infection or vaccination.

The model includes parameters to quantify duration and degree of infectiousness; duration and degree of natural, vaccine, and maternally acquired immunity; rates of community mixing between age groups; and transmission coefficients for the household and community settings. Parameter values for each individual are selected from the distributions described in Table 1. The duration of infectiousness for cases in naive and primed individuals has a mean of 21 days, consistent with published values [19]. The contribution of infections in primed individuals to the force of infection is yet to be convincingly resolved for pertussis [7, 20]; we assume infections arising in susceptible primed individuals are half as infectious as those in naive individuals.

**Table 1.** Parameter Distributions for the Epidemiologic Model

Parameter	Distribution Type	Mean/Fixed Value
Duration of infectiousness (naive and primed)	Gamma Shape parameter = 3	3 wk
Infectiousness in primed individuals relative to naive	Fixed value	0.5
Duration of full immunity (full immunity states [natural and vaccine])	Exponential	9 mo
Duration of partial immunity (partial immunity [natural] state)	Exponential	74 y
Duration of partial immunity following DTP3 vaccination (partial immunity [vaccine] state)	Exponential	4 y
Duration of partial immunity following antenatal vaccination (partial immunity [vaccine] state)	Exponential	6 y
Susceptibility in primed individuals relative to naive	Fixed value	0.6
Duration of maternally acquired immunity	Gamma Shape parameter = 3	12 wk

We assume recently exposed or vaccinated individuals have a short duration of full immunity (full immunity; sampled from an exponential distribution with mean 9 months) followed by a much longer duration of partial immunity (partial immunity) [7]. We assume differences in the mean duration of partial immunity to infection following exposure and booster vaccination of an order of magnitude (74 and 6 years, respectively), consistent with the findings of previous modeling studies [7, 21]. We assume a duration of protection following the full primary course of vaccination of 4 years, slightly less than that of booster doses. Based on our previous compartmental modelling, we assume susceptible individuals primed by infection or vaccination (susceptible primed) are 0.6 times as likely to be infected as those naively susceptible [7].

#### Maternal and Infant Immunity

Infants born to mothers with full or partial immunity, acquired from natural infection or vaccination, start their lives fully protected against infection (maternal protection) by antibodies acquired from their mothers. Once these passive antibodies have waned below a threshold level, infants become fully susceptible to infection (susceptible naive). Although passive antibodies against pertussis wane rapidly, with the starting level likely related to the time elapsed since a mother's natural infection or vaccination [22], in previous work our results were relatively insensitive to durations of passive protection ranging from 6 to 24 weeks [8]. Thus, here we sampled the duration of maternally acquired immunity from a distribution with a mean of 12 weeks. Infants born to nonimmune mothers start their lives naively susceptible to infection.

#### Application of Childhood Vaccination in the Model

Our previous pertussis modeling showed the importance of the duration of natural immunity and historical vaccine coverage in driving long-term pertussis trends [7]. We therefore expected that, for each setting, the time since introduction of infant vaccination and coverage achieved would influence the impact of an antenatal pertussis program. While many LMICs adopted diphtheria-tetanus-pertussis (DTP) vaccines as part of the EPI in the late 1970s to mid-1980s (eg, The Gambia; Senegal) [4, 23, 24], uptake in other countries has been slower (eg, Nigeria) [25]. The percentage of 1-year-olds receiving the full primary course in LMICs is heterogeneous, in 2011 ranging from very low levels in some countries, for example, Chad (22%), through low levels in Nigeria (47%) and Ethiopia (51%) to >80% in many countries such as Kenya (88%) [26]. To provide for the wide range of vaccination experiences across LMICs, in this study we simulated 9 combinations of vaccination start year and coverage. We allowed for infant vaccination to commence in 1985, 1995, or 2005 and for each of these implementation dates, we increased infant coverage linearly from 0% in the year of introduction to 20%, 50%, or 80% after 8 years, with coverage remaining constant thereafter. While recognizing that real coverage rates are subject to variation due to a number of factors, our selected combinations cover a broad range of population immunity profiles.

We applied routine infant vaccination as a primary course at 2, 4, and 6 months, assuming that infants either receive all 3 primary doses on time, or none, hereafter referred to as three doses of diphtheria, tetanus, and pertussis vaccines (DTP3). Infants successfully vaccinated obtained full immunity, with primary vaccine failures retaining their naive susceptible state. The probabilities of successful vaccination after each dose were calculated such that 53%, 81%, and 85% of vaccine recipients were protected against infection after 1, 2, and 3 doses, respectively, following our previous model [8] and described in the Supplementary Materials. Childhood boosters were not applied in the model due to limited availability and variable uptake in LMICs [2].

### Simulation of Antenatal Vaccination Programs

Simulations of the model for each of the combinations of DTP3 start year and coverage were run until the end of model year 2014, providing 9 different initial conditions for which we investigated the impact of antenatal vaccination. For each of the 9 initial conditions, 5 different antenatal vaccination scenarios were simulated (20 simulation runs for each initial condition/scenario combination), commencing in model year 2015 and continuing for 10 years:

1. Baseline (infant schedule at 2, 4, and 6 months; continue previous coverage);
2. Baseline + antenatal vaccination (single-dose eligibility; maternal coverage equivalent to DTP3);
3. Baseline + antenatal vaccination (multidose eligibility; maternal coverage equivalent to DTP3);
4. Baseline + antenatal vaccination (single-dose eligibility; maternal coverage greater than DTP3);
5. Baseline + antenatal vaccination (multidose eligibility; maternal coverage greater than DTP3);

In the “single-dose eligibility” scenarios, a mother is eligible to receive a single dose after the introduction of the antenatal program; in the “multidose eligibility” scenarios, for each birth after the introduction of the antenatal program a mother is eligible to receive a vaccination dose. For the “maternal coverage greater than infant” scenarios, maternal coverage was set to 60%, 75%, or 90% for DTP3 coverage of 20%, 50%, and 80%, respectively.

For each of the 9 initial conditions, first we report infection incidence over the period 2015–2024 in children aged 0–2 months and 0–1 year under baseline conditions (scenario 1). Second, we report infection incidence over the period 2015–2024 in these same age groups under each of the antenatal vaccination strategies (scenarios 2–5). We expect the incidence of infection to closely reflect the disease burden in these age groups, as these are first pertussis infections occurring at an early age and are therefore highly likely to be symptomatic.

## RESULTS

### Effect of Childhood Vaccination Program

Prior to the implementation of DTP3, model-generated incidence patterns for exemplar stochastic realizations of the model showed epidemics every 3–4 years, incidence rates in naive individuals approximating the size of the birth cohort, and 95% of individuals experiencing their first infection before the age of 10.

We compared, over a 10-year period, the impact of 9 alternative DTP3 coverage/start year combinations. The impact of our baseline vaccination scenario (DTP3 at 2, 4, and 6 months) on annual infection incidence in infants aged 0–2 months averaged over the period 2015–2024 is shown in Supplementary Figure 1, and for those aged 0–1 year in Supplementary Figure 2. Incidence over this period reduced with increasing DTP3 coverage, regardless of the year of introduction. For moderate and high DTP3 coverage (50% and 80%, respectively), incidence was lower when introduction occurred later. Year of DTP3 introduction did not affect incidence under our baseline scenario when coverage was low (20%).

In our model, the introduction of a childhood vaccination program had little effect on the proportion of infants born with antibody passively acquired from their mother as a result of exposure to *Bordetella pertussis*. Across our 9 combinations of vaccination start year and vaccination coverage, this proportion varied minimally from 0.88 (interquartile range [IQR], 0.88–0.88) for 80% DTP3 coverage introduced in 1985 to 0.94 (IQR, 0.94–0.94) for 20% DTP3 coverage introduced in 2005 (Table 2). This maintenance of infant passive protection resulted from the continued high circulation of pertussis in older age groups, providing opportunities for regular boosting of adult immunity.

**Table 2.** Proportion of Infants Born With Passive Protection Acquired From Their Mothers, With DTP3 and No Antenatal Vaccination in Place

DTP3 Coverage	Start Year		
	1985	1995	2005
20%	0.93 (0.93–0.93)	0.93 (0.93–0.93)	0.94 (0.94–0.94)
50%	0.91 (0.91–0.91)	0.92 (0.91–0.92)	0.94 (0.94–0.94)
80%	0.88 (0.88–0.88)	0.90 (0.89–0.90)	0.91 (0.91–0.91)

Data are presented as median (interquartile range).

Abbreviation: DTP3, three doses of diphtheria, tetanus, and pertussis vaccines.

### Impact of Antenatal Vaccination

For each of our DTP3 start year/coverage initial conditions, we compared the impact over a 10-year period of antenatal vaccination delivered for the “single-dose eligibility” and “multidose eligibility” scenarios at equivalent or greater coverage relative to DTP3 coverage. The incidence of infection in 2 age groups, 0–2 months and 0–1 year, was considered to assess the ability of antenatal vaccination: (1) to protect infants too young to be vaccinated (0–2 months) through provision of passive protection; and (2) to reduce the overall burden of infant disease (0–1 year) through indirect protection of infants and other household members due to the mother's immunity.

**Figure 2.**
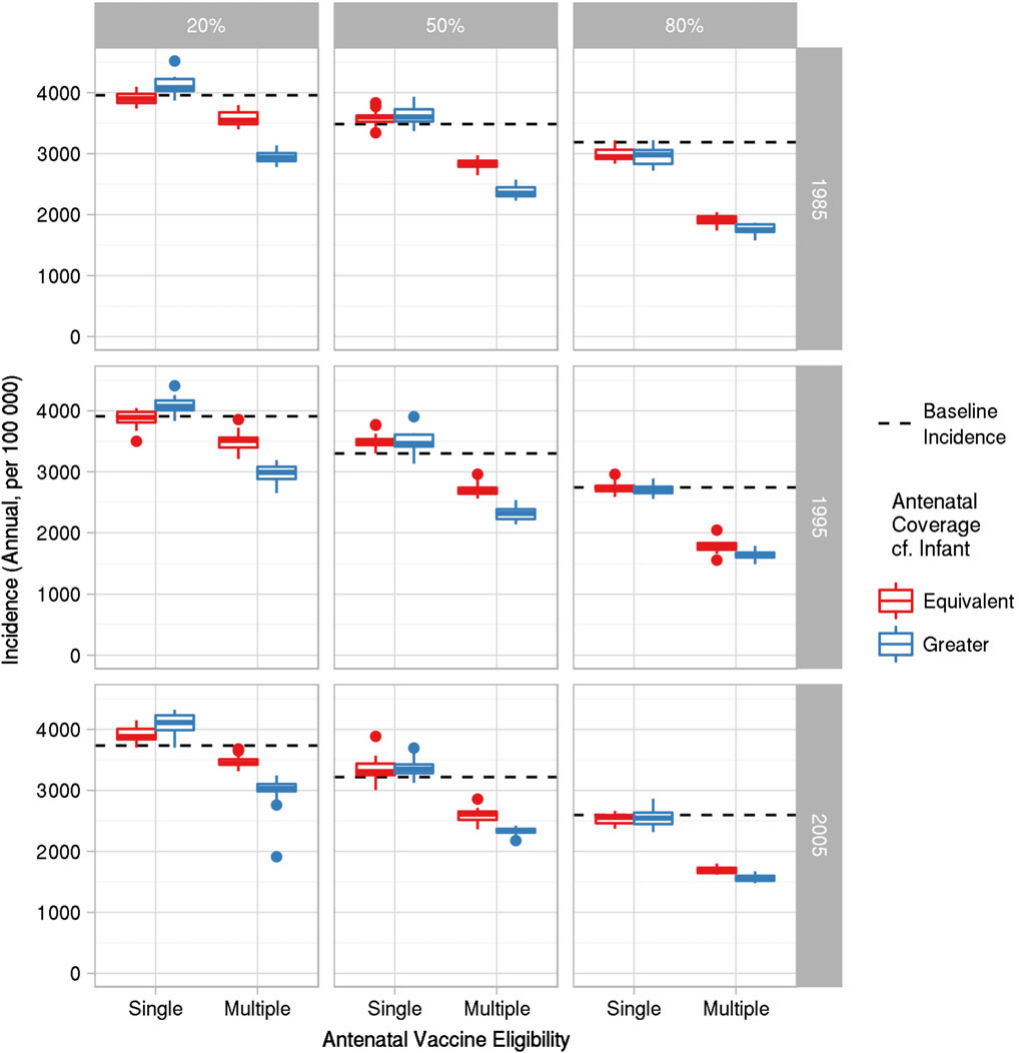
Annual infection incidence in 0- to 2-month-olds averaged over 2015–2024 for single- and multidose antenatal vaccination eligibility and coverage equivalent to, or greater than, that for three doses of diphtheria, tetanus, and pertussis vaccines (DTP3). Rows represent year of DTP3 introduction; columns represent DTP3 coverage.

For infants too young to be vaccinated (0–2 months), the provision of a “single-dose eligibility” antenatal vaccination program produced a minimal impact on infection incidence compared to baseline for all DTP3 initial conditions, irrespective of whether antenatal vaccination was delivered at equivalent or greater coverage relative to DTP3 (Figure 2). By contrast, a “multidose eligibility” antenatal vaccination strategy reduced incidence for these infants under all DTP3 initial conditions, with larger decreases as DTP3 coverage (and thus antenatal coverage also) increased, regardless of vaccination start year (Figure 2). When antenatal vaccination was applied at a greater coverage than DTP3 vaccination, incidence was further reduced for initial conditions with low or medium DTP3 coverage.

**Figure 3.**
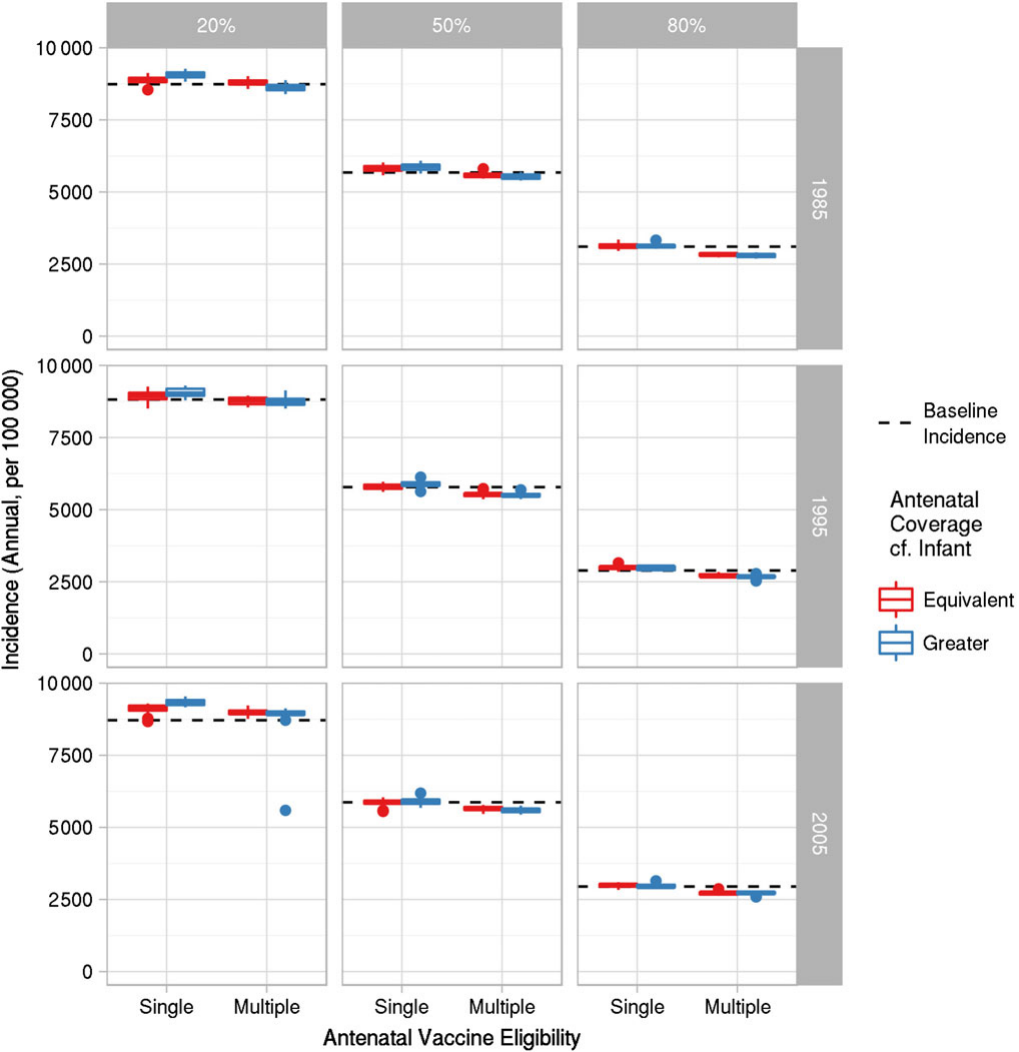
Annual infection incidence in 0- to 1-year-olds averaged over 2015–2024 for single- and multidose antenatal vaccination eligibility; and coverage equivalent to, or greater than, that for three doses of diphtheria, tetanus, and pertussis vaccines (DTP3). Rows represent year of DTP3 introduction; columns represent DTP3 coverage.

Similarly, across all infants aged <1 year, a “single-dose eligibility” antenatal vaccination strategy had little effect on infection incidence compared to baseline over all DTP3 initial conditions (Figure 3). Over this time frame, even when mothers were eligible for multiple antenatal doses, reductions in incidence compared to baseline were slight (around 5%), even when antenatal coverage was greater than DTP3 coverage.

**Figure 4.**
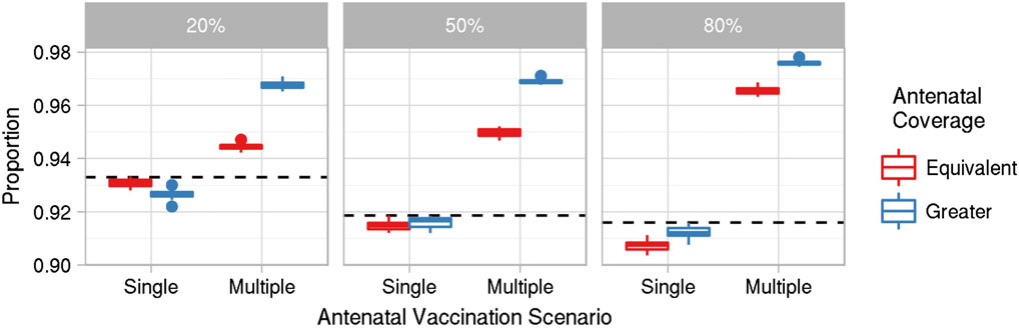
Proportion of infants born with maternal protection over the period 2015–2024 for single- and multidose antenatal vaccination eligibility (dotted line shows results under three doses of diphtheria, tetanus, and pertussis vaccines (DTP3) only); and coverage equivalent to, or greater than, DTP3, where DTP3 vaccination was introduced in 1985. Note that the y-axis does not start at 0, in order to more clearly show the differences between strategies.

While an antenatal strategy in which mothers were eligible to receive only a single dose had little effect on the proportion of infants born with passive immunity, eligibility for multiple antenatal doses increased this proportion, compared with having DTP3 only in place (Figure 4).

## DISCUSSION

Using an individual-based model of pertussis transmission, we found that antenatal vaccination can reduce infection among infants aged 0–2 months in settings characterized by high population growth rates and large household sizes. More pronounced reductions could be achieved if mothers are eligible for multiple antenatal doses. Introducing an antenatal vaccination program in LMICs could thus potentially reduce infant mortality by preventing infection among the most vulnerable 0- to 2-month age group. Despite this reduction of infection risk in the 0- to 2-month age group, across the full first year of life antenatal vaccination has virtually no impact on the incidence of infection, even when administered at greater coverage than DTP3. For infants aged 0–1 year, the direct protection provided by DTP3 acts more strongly to reduce infection incidence than the short-lived protection provided by maternal antibodies. We therefore anticipate that in LMICs, broader gains at the population level would be achieved by focusing efforts on increasing DTP3 coverage than implementing an antenatal vaccination program, particularly if severe morbidity (and associated mortality) extends beyond the first 2 months of life.

Pertussis circulation has remained high in many LMICs, due to the recency of vaccine introduction and limited DTP3 coverage. In such settings, a high proportion of infants are likely to be born with maternal protection derived from their mother's own prior infection or immunity-boosting exposure. Therefore, there are relatively few infants who stand to benefit from the passive protection provided by antenatal vaccination compared with settings in which high vaccine coverage has been longstanding. When we examined the impact of antenatal pertussis vaccination in Australia, the predicted reduction in infection incidence was much more pronounced, due to the substantially lower proportion of infants born without passive protection [8]. As DTP3 coverage increases in LMICs, with a consequent decrease in circulation, we could begin to see cohorts of women, vaccinated as newborns, who have never been infected. As these women become mothers, their pertussis antibody levels might be insufficient to provide passive protection to their infants, as may have occurred in high-coverage settings. Estimation of the degree to which pertussis vaccination has interrupted transmission in LMICs is complex, as we are only just beginning to see cohorts of adults for whom their first experience of pertussis was vaccination rather than infection.

To anticipate a reduction in the proportion of infants born without maternal protection—a condition that favors antenatal vaccination—it is important to monitor pertussis in LMICs for signs of a reduction in circulation. Monitoring of seroprevalence at a population level, particularly among women of childbearing age, is one possible way of assessing epidemiologic trends. Reduced antibody levels, suggestive of reduced circulation, would logically lead to a decrease in the proportion of neonates born with passive protection.

Our model considers a population representative of a high fertility setting under a range of idealized historic DTP3 schedules. There is a great deal of demographic and epidemiologic diversity across LMICs, and the conclusions of our work need to be interpreted in this light. In previous work, we found that changes in vaccination schedule and coverage are important drivers of pertussis epidemiology [7]. For our model to better reflect the pertussis experience of a particular country, more detailed information on historical demographic and vaccination coverage trends would need to be incorporated.

Limited population-wide surveillance data for pertussis in LMICs makes validation of our model challenging. Other than research studies undertaken in small, intensively studied populations, such as Senegal [23], many published studies report pertussis prevalence in hospitalized patients or patients with persistent cough, unlikely to accurately estimate pertussis prevalence in the whole population [27–29]. In Senegal, as EPI coverage increased from 13% in 1986 to 82%–84% after 1990, cases in infants aged <2 months decreased from around 200 per 1000 to around 56 per 1000 [23]. Although this is a somewhat larger reduction than the roughly 30% we observed in our model upon introduction of DTP3 at 80%, it is pleasing to note that model-generated incidence rates after the introduction of DTP3 are of the same order of magnitude as this published study [23].

As with every modeling study, there are some necessary limitations to our work. First, we consider infection in infants <1 year of age, which we assume to be indicative of the disease burden in this age group. We do not quantify the incidence of infection in children aged >1 year, as increasing age and prior immunity to pertussis are known to complicate the relationship between pertussis infection and disease. While the disease burden in older age groups may still be quite important in LMICs, we prioritized younger age groups, as the very young are more vulnerable to serious consequences of pertussis infection and death.

Second, we have not conducted formal sensitivity analyses around our infection and disease assumptions, as our prior modeling work showed that the impact of antenatal vaccination was generally robust to these assumptions [8]. As in our previous publication, we have modeled a vaccine with a duration of protection substantially lower than the duration of protection provided by natural infection. Were vaccine immunity to persist over a longer timescale, the effectiveness of the “single-dose” strategy may be increased. However, given the extended span of childbearing years (85% of births are for mothers aged 15–35 years) in high-fertility settings, this protection would need to be robust over at least 2 decades. While we assume that passive protection derived from mothers whose immunity results from natural infection and boosting of immunity through exposure is equivalent to that following vaccination, some uncertainty exists around this assumption [22].

Finally, we have not included any childhood booster vaccinations in our model due to the considerable heterogeneity of availability and coverage in LMICs. It is important to consider the possibility that antenatal vaccination may blunt an infant's response to the DTP3 schedule [30], which may have implications in countries that do not provide a booster in early life.

Our work demonstrates the importance of considering local context and drivers of disease in implementation of vaccine programs. Historical vaccination coverage and the longevity of the vaccine program need to be taken into account when estimating the likely impact of introducing antenatal booster vaccinations. The model supports further assessment of maternal seroprotection as an indicator of likely program success.

## Supplementary Data

Supplementary materials are available at http://cid.oxfordjournals.org. Consisting of data provided by the author to benefit the reader, the posted materials are not copyedited and are the sole responsibility of the author, so questions or comments should be addressed to the author.

Supplementary Data
