# Supplementary material for: Influence of Population Demography and Immunization History on the Impact of an Antenatal Pertussis Program
Source: Clin Infect Dis. 2016 Nov 2;63(Suppl 4):S213–20. doi: 10.1093/cid/ciw520 (PMC5106613; doi:10.1093/cid/ciw520)
Supplement: Supplementary Data [file supp_63_suppl-4_S213__index.html]

Supplementary Data 

# Influence of Population Demography and Immunization History on the Impact of an Antenatal Pertussis Program

## Supplementary Data

Supplementary Data

- Supplementary Data - Pdf file
