# Supplementary material for: Influence of Population Demography and Immunization History on the Impact of an Antenatal Pertussis Program
Source: Clin Infect Dis. 2016 Nov 2;63(Suppl 4):S213–20. doi: 10.1093/cid/ciw520 (PMC5106613; doi:10.1093/cid/ciw520)
Supplement: Supplementary Data [file supp_ciw520_ciw520supp.pdf]

Supplementary Material

Influence of population demography and immunization history on the impact of an antenatal pertussis program

Patricia Therese Campbell, Jodie McVernon, Peter McIntyre, Nicholas Geard

1 Outline of the demographic model

We use an individual based model, in which each individual is characterised by age, sex, household of residence, and family ties. Over time, individuals are born, age, form and dissolve couples and household units, and die, with probabilities determined by their sex, current age and life stage. The population demography is updated at each time step of the simulation (in the simulations reported here, at weekly intervals) according to the following procedure:

1. The age of each individual is incremented by the appropriate number of days.

2. For each individual i, one of the following may occur:

    (a) **Death:** with a probability based on i's age and sex, i dies and is removed from the population. An individual j is chosen to be the mother of a replacement individual as follows:

    i. The target age of the mother is determined on the basis of age-specific fertility rates.

    ii. A set of candidate mothers is determined on the basis of age, eligibility to give birth (*i.e.*, not having given birth in the previous 9 month period) and household status (for simplicity, individuals are not eligible to give birth while living with their own parents).

    iii. j is selected at random from the pool of candidate mothers.

    If the death of i results in a household containing only children, these individuals are reallocated as follows:

    i. Any children aged 18 or older form new single-person households.

    ii. Any children aged less than 18 are randomly allocated (fostered) to other households containing at least one child.

(b) **Couple formation:** if i is currently single, with a probability based on i's age, i forms a couple with an individual j, chosen as follows:

i. The target age of the partner j is determined on the basis of i's age.

ii. A set of candidate partners is determined on the basis of age, sex, and not currently being a member of a couple.

iii. j is selected at random from the pool of candidate partners.

The households of i and j are merged (along with any children currently residing with them) or, if both previously lived with their parents, a new household of size two is created.

(c) **Leaving home:** if i is currently living with their parents, with a probability based on i's age, i leaves their parents' household and forms a new household of size one.

(d) **Couple separation:** if i is currently in a couple, with a probability based on i's age, i separates from that couple and forms a new household; for simplicity, we assume that any children residing with the couple when they separate join the mother's household.

3. To simulate a growing (or shrinking) population, the number of additional births (or deaths) required to occur in the current time step to match the target growth rate is calculated and additional birth (or death) events are triggered.

1.1 Population model parameters and data sources

We have calibrated our demographic model to produce a hypothetical low income country population characterised by (compared to a more developed country):

- a lower median age of approximately 15 years;
- a larger mean household size of approximately 5 individuals;
- a higher growth rate due to natural increase of 2.5% per year;
- a lower life expectancy of approximately 52 years;
- a lower age of first childbirth: 15% of women have had their first birth by 18 years, and median age of women at childbirth is 24 years.

To achieve this, we used age-specific mortality and fertility rates for Zambia (sourced from `http://data.un.org`), a sub-Saharan country with a medium-low Human Development Index. However, our population and results should not be interpreted as being specific to Zambia, as we have not attempted to capture all potentially relevant characteristics of the country's demographic trajectory.

Parameters for household transitions (couple formation and separation, leaving home) were estimated on the basis of reported estimates that 40–50% of women are married by the age of 18

(http://zambia.unfpa.org/), and calibrated to observed household size distributions. We assume that individuals become eligible to leave their parents' household, either independently or as a member of a couple, at 15 years. As a consequence, individuals also become eligible to separate from a couple at 15 years. The annual probability of a single individual entering a couple is 0.08. The annual probability of a couple separating is 0.005. The annual probability of a single individual leaving home and forming a one person household is 0.05.

2 Outline of the epidemiological model

The epidemiological model extends the demographic model described above, tracking the current disease state of each individual according to the state transition model (Figure 1 in the main body of the paper). The disease state of the population is updated at each time step of the simulation (in the simulations reported here, at weekly intervals) according to the following procedure:

1. Population demography is updated, as described in Section 1.
2. Periodically (at 5 year intervals in the simulations reported here), the community contact matrix (see below for detail) is updated to account for the changing age structure of the population.
3. Individuals born in the current time step are assigned the **maternal protection** state if their mother is currently in the **full immunity** or **partial immunity** state, otherwise they are assigned the **susceptible (naïve)** disease state.
4. Individuals eligible for vaccination on the basis of their age and previous vaccine status are vaccinated with time- and scenario-dependent probabilities as described below. Vaccinated individuals are assigned the **full immunity** state.
5. For each individual who is currently **susceptible** to infection (naïve), the force of infection acting on that individual is calculated according to Equation 1, and used to specify the probability of that individual becoming **infected** in the current time step.
6. For each individual who is currently **susceptible** to infection (primed), the force of infection acting on that individual is calculated according to Equation 1, reduced by a factor σ, and used to specify the probability of that individual becoming **infected** in the current time step.
7. The set of individuals who are exposed to infection is determined stochastically; this includes both **susceptible** individuals who will become **infected**, and **full immunity** or **partial immunity** individuals who will have their protection boosted.
8. The disease states of all individuals are updated simultaneously; that is, the state of the population at time $t + 1$ is determined by the state of the population at time t. Newly **infected** individuals are assigned a counter corresponding to their infectious duration as described below.

Table S1: Parameter distributions for the epidemiologic model.

Parameter	Distribution type	Mean/Fixed value
Duration of infectiousness (naïve and primed)	Gamma	3 weeks
Infectiousness in primed individuals relative to naïve	Fixed value	0.5
Duration in Full immunity state	Exponential	9 months
Duration in Partial immunity state – following infection	Exponential	74 years
Duration in Partial immunity state – following DTP3 course	Exponential	4 years
Duration of in Partial immunity state – following antenatal vaccination	Exponential	6 years
Susceptibility in primed individuals relative to naïve	Fixed value	0.6
Duration of maternally acquired immunity	Gamma	12 weeks
Household transmission coefficient, q_h	Fixed value	0.8
Community transmission coefficient, q_c	Fixed value	0.01

9. Individuals in the states **maternal protection**, **infected**, **full immunity** or **partial immunity** have their relevant counter decremented. Individuals whose counter reaches zero will attain the **full immunity** state if they were previously **infected**, **partial immunity** if they were previously in the **full immunity** state or **susceptible (primed)** if they were previously in the **partial immunity** state.

The length of time that an individual remains in the **maternal protection** or **infected** states is drawn at random from a Gamma distribution with shape parameter $k = 3$ and mean parameter μ specified in Table S1. The length of time that an individual remains in the **full immunity** or **partial immunity** states is drawn at random from an exponential distribution with mean specified in Table S1.

Note that a single set of **full immunity** and **partial immunity** states are used to capture protection arising from both first and subsequent infections, and from primary course and booster vaccinations. However, the time duration that an individual remains in that state will vary depending on the source of that protection. When a currently protected individual has their immunity boosted (as a result of vaccination or exposure), we enforce a rule that they can never have their duration of protection reduced. For example, an individual who has 35 years of protection as a result of natural infection will not have their duration of protection reduced if they are subsequently vaccinated.

2.1 Household and community transmission parameters

The probability of a susceptible person, p, in age class i becoming infected in a given time period is a combination of the prevalence of infection in their household, and in the broader community, weighted by the age-specific patterns of contact in the community. We used the same method to derive the contact matrix as in our previous work [2]. The per time step probability of infection of person $p = 1 - e^{-\lambda_p(t)\Delta t}$, where

$$\lambda_p(t) = \sum_{k \in H} \zeta \frac{q_h I_k(t)}{N_H(t) - 1} + \sum_j \eta_{ij} \frac{q_c I_j(t)}{N_j(t)}, \tag{1}$$

where H is p's household, $N_H(t)$ is the number of people in H, k is a housemate of p, ζ is the number of effective contacts per day between p and their housemates (here, $\zeta = 1$), $I_k(t) = 1$ if k is infectious and 0 otherwise, q_h is the per contact household transmission coefficient, i is the age of p, η_{ij} is the mean number of contacts in the community per day between people of age i and people of age j, q_c is the per contact community transmission coefficient, $I_j(t)$ is the number of infectious people of age j and $N_j(t)$ is the total number of people of age j.

Within the community, we make the standard assumption for large populations that transmission is frequency dependent. The degree to which transmission within the household is frequency or density dependent is not well-established, but for a highly transmissible pathogen such as pertussis, the resulting dynamics are relatively invariant [3], and the results reported here use frequency dependent household transmission.

3 Vaccination dose response

The probabilities for success at each primary course dose were calculated to achieve effective protection after 1, 2 and 3 doses of 53%, 81% and 85% of vaccine recipients respectively, based on published vaccine effectiveness values [4]. We apply a moderate probability of 0.13 for antenatal booster failure against infection, consistent with booster responses at different ages [1], and make the simplifying assumption booster response is independent of primary course response. As a general principle, individuals cannot have their duration of immunity reduced as a result of successive exposures or vaccination.

4 Additional results

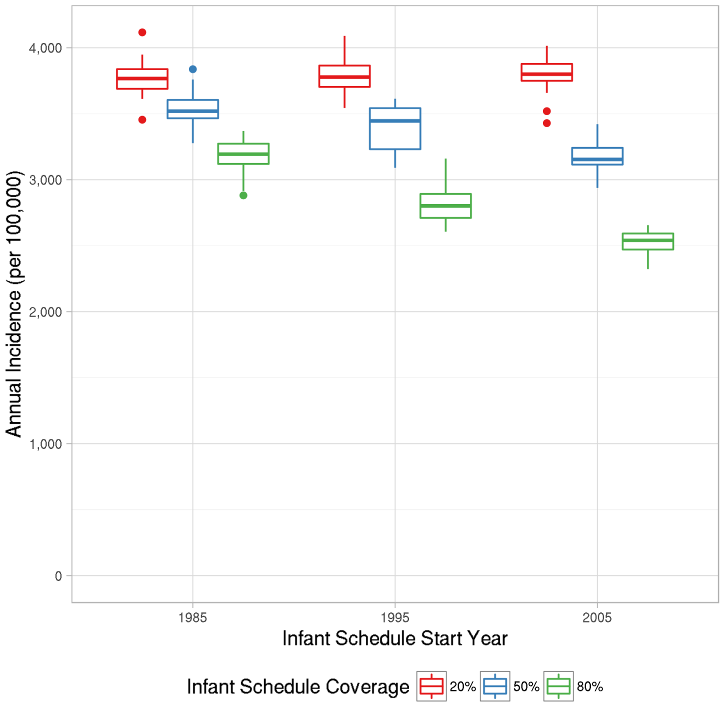

Figure S1: Annual infection incidence in 0–2 month old infants with a DTP3 schedule in place (baseline), averaged over a 10 year period from 2015-2024. Model generated incidence, summarised over all simulation runs are shown for each DTP3 coverage/start year combination.

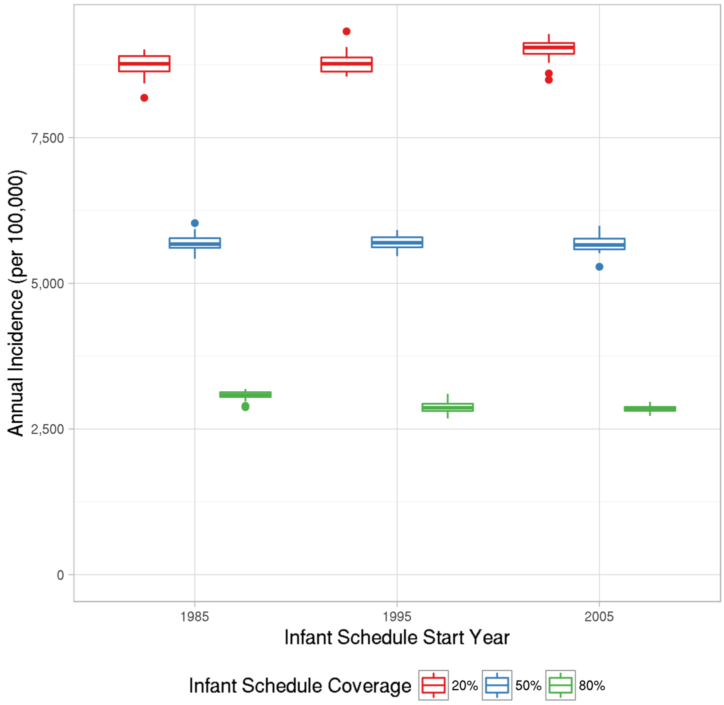

Figure S2: Annual infection incidence in 0–1 year old infants with a DTP3 schedule in place (baseline), averaged over a 10 year period from 2015-2024. Model generated incidence, summarised over all simulation runs are shown for each DTP3 coverage/start year combination.

References

[1] Berner, R., Boisnard, F., Thomas, S., Mwawasi, G., and Reynolds, D. (2012). Safety and immunogenicity of fully liquid DTaP(5)-IPV-Hib pediatric combination vaccine (Pediacel) compared to DTaP(3)-HBV-IPV/Hib (Infanrix Hexa) when coadministered with heptavalent pneumococcal conjugate vaccine (PCV7) as a booster at 11-18 months of a. *Vaccine*, 30(35):5270–5277.

[2] Campbell, P. T., McVernon, J., and Geard, N. (in press). Determining best strategies for maternally-targeted pertussis vaccination using an Individual-Based Model. *American Journal of Epidemiology*.

[3] Geard, N., Glass, K., McCaw, J. M., McBryde, E. S., Korb, K. B., Keeling, M. J., and McVernon, J. (2015). The effects of demographic change on disease transmission and vaccine impact in a household structured population. *Epidemics*, 13:56–64.

[4] Quinn, H. E., Snelling, T. L., Macartney, K. K., and McIntyre, P. B. (2014). Duration of protection after first dose of acellular pertussis vaccine in infants. *Pediatrics*, 133(3):e513–9.
